# Therapeutic Use of Stem Cells for Myocardial Infarction

**DOI:** 10.3390/bioengineering5020028

**Published:** 2018-04-06

**Authors:** Mariah Madigan, Rony Atoui

**Affiliations:** 1 Northern Ontario School of Medicine, Sudbury, ON P3E 2C6, Canada; mariahmadigan@icloud.com; 2 Health Sciences North, Sudbury, ON P3E 5J1, Canada

**Keywords:** stem cell therapy, myocardial infarction, bone marrow, mesenchymal stem cells, hematopoietic stem cells, embryonic stem cells, induced pluripotent stem cells, cardiac stem cells

## Abstract

Myocardial infarction is a leading cause of morbidity and mortality worldwide. Although medical and surgical treatments can significantly improve patient outcomes, no treatment currently available is able to generate new contractile tissue or reverse ischemic myocardium. Driven by the recent/novel understanding that regenerative processes do exist in the myocardium—tissue previously thought not to possess regenerative properties—the use of stem cells has emerged as a promising therapeutic approach with high expectations. The literature describes the use of cells from various sources, categorizing them as either embryonic, induced pluripotent, or adult/tissue stem cells (mesenchymal, hematopoietic, skeletal myoblasts, cardiac stem cells). Many publications show the successful use of these cells to regenerate damaged myocardium in both animal and human models; however, more studies are needed to directly compare cells of various origins in efforts to draw conclusions on the ideal source. Although numerous challenges exist in this developing area of research and clinical practice, prospects are encouraging. The following aims to provide a concise review outlining the different types of stem cells used in patients after myocardial infarction.

## 1. Introduction

Cardiovascular disease (CVD) is the leading cause of morbidity and mortality worldwide, with acute myocardial infarction (AMI) representing the most common CVD [1]. Coronary artery disease (CAD) resulting in myocardial infarction (MI) can lead to ischemic heart damage, which, depending on the ability and timing of reperfusion, may be reversible or irreversible [2]. Cardiomyocytes depend on constant delivery of oxygen and nutrients due to the heart’s limited capacity for anaerobic metabolism [3]. Ischemic heart damage disrupts this homeostatic balance, which leads to a series of cellular events including apoptosis, necrosis, various inflammatory responses, and remodeling processes resulting in scar formation, ventricular stiffness, and altered contractibility [3]. Though medical and surgical treatments such as pharmacotherapy, percutaneous coronary intervention (PCI), and surgical management can significantly improve patient outcomes and reverse viable ischemic tissue, no current treatment is able to create new contractile tissue or regenerate lost myocardium [4]. Heart transplantation is the only definitive treatment for the ischemic heart failure that results after MI; however, the procedure involves significant risk and donors are limited [5].

Therefore, driven by the relatively novel understanding that regenerative processes do exist in the myocardium—tissue previously thought to be post-mitotic—the use of stem cells has emerged as an encouraging therapeutic approach with high expectations for patients with limited/no other treatment options [5,6].

Stem cells have two distinct properties underlying their use in the field of regenerative medicine: their capacity for self-renewal, and their ability to differentiate into a variety of tissues (known as potency) [7]. Cells can be unipotent, able to differentiate into only one cell type; multipotent, able to differentiate into a limited number of cell types; or pluripotent, able to differentiate into all cell types that make up the human body [5].

To improve cardiac function, an ideal cell therapy should generate a new vascular network and new contractile tissue that can align and synchronize with existing heart tissue; in addition, cells must be able to differentiate into cardiac lineages (i.e., myocytes, vascular endothelial cells), or act via paracrine mechanisms to promote regenerative processes [6]. The optimal cell type should have feasible extraction, isolation, expansion, and safe and effective delivery into humans. The search for the optimal cell type has led to the study of a variety of cell populations and sources, as well as delivery methods, each with individual advantages and disadvantages [8].

The literature describes the use of numerous types of cells from various sources (skeletal myoblasts, bone marrow, hematopoietic, mesenchymal, embryonic, induced pluripotent, cardiac stem cells). Many publications show the successful use of these cells to regenerate damaged myocardium in both animal and human models; however, conflicting results exist, procedural characteristics lack standardization, and more studies are needed to directly compare cells in efforts to draw conclusions on the ideal source. Although numerous challenges exist in this developing area of research and clinical practice, prospects remain encouraging.

The following review aims to provide a summary of the primary sources of stem cells used in the treatment of MI.

## 2. Materials and Methods 

A PubMed search was done using search terms such as “stem cell therapy for myocardial infarction”, “(stem cells) AND myocardial infarction”, “(Mesenchymal stem cells) AND myocardial infarction”, “(hematopoietic stem cells) AND myocardial infarction”, and “(induced pluripotent stem cells) AND myocardial infarction”. The secondary search strategy included cross-referencing articles from primary resources. Publication date was restricted to the last 5 years using the PubMed search parameters feature. No publication status restrictions were imposed. The first search was run on 16 December 2017, and the last search was run on 31 January 2018. Types of studies analyzed included reviews, systematic reviews, meta-analyses, and clinical trials reporting the use of stem cell therapy for myocardial infarctions. Language was restricted to publications in English.

## 3. Results

### 3.1. Skeletal Myoblasts

Skeletal myoblasts were the first cell type discovered to have potential for use in the treatment of CVD in both animals and humans [9]. These progenitor cells are abundant in the human body, have myogenic properties, are capable of in vitro proliferation/expansion, have contractile abilities, and have the capacity to resist ischemic insult [10].

Following successful animal studies, the MAGIC study was the first clinical trial using myoblast stem cell transplantation [11]. Table 1 outlines the major clinical trials involving various stem cell sources used in the treatment of MI's. Results did not match those found in animal models, showing no evidence of improved left ventricular (LV) function when compared with standard coronary artery bypass graft (CABG) without the addition of stem cell transplantation. Further studies found similar results, failing to achieve differentiation into cardiomyocytes, and contributing to incidence of new arrhythmias, concluding that skeletal myoblasts are committed to the skeletal muscle lineage [10]. Some researchers aimed to overcome limitations of functional electromechanical integration by introducing a gap junction protein (connexin 43) through genetic modification; however, this did not prove successful [12].

### 3.2. Bone Marrow Stem Cells

In 2001, it was first shown that there was potential for infarcted myocardium to be regenerated with the transplantation of bone-marrow-derived stem cells (BMSCs) [13]. Autologous bone marrow mononuclear stem cells (BMMNCs) were one of the first tested in clinical settings, and to date have been the cell type used most frequently in clinical trials for treating CVD [14,15]. BMMNCs are a heterogeneous population including monocytes, lymphocytes, mesenchymal stem cells (MSCs), hematopoietic stem cells (HSCs), and endothelial progenitor cells [14].

After the first documented use, small phase I trials demonstrated the therapy to be feasible and safe [16,17]. Early studies showed promising results with patients experiencing beneficial cardiac effects including enhanced perfusion, increased regional or global left ventricular ejection fraction (LVEF), and reduced LV end systolic volumes (LVESV) [15]. Following early studies, clinical trials emerged, though patient numbers have remained small [18,19].

A 2012 review by the Cochrane Heart Group analyzed data from 33 clinical trials reporting on BMSC therapy post-AMI. Findings showed lasting improvement in LVEF in follow-up periods ranging from 12 to 61 months, although this did not translate to decreased morbidity/mortality [20]. In contrast, the group also published a 2014 meta-analysis of 23 clinical trials involving BMSC therapy in ischemic heart disease (IHD) and congestive heart failure (CHF) patients, which concluded that improved LVEF did translate to decreased morbidity and mortality [21].

A 2017 systematic review outlines the timeline of developments in BMMNC therapy over the last 15 years [14]. They report on a number of studies that have yielded promising results. The BOOST trial showed a 6.7% improvement in LV function compared with 0.7% in the control group, though at 18 month follow-up there was no longer a difference in LVEF between groups, a finding that remained present at 5 years [16]. REPAIR-AMI was the first trial to evaluate timing of cell delivery, finding that intracoronary cell transplantation 5 or more days after PCI was associated with more significant effects than procedures performed at 4 days or less, which displayed minimal LVEF improvement [19]. Contrary to BOOST trial findings, improvement in regional LV contractility of infarcted segments was present at a 2 year follow-up [22]. Additionally, their combined endpoint of death, recurrent hospitalization for heart failure, and repeat MI was reduced in the treatment group compared with the placebo at a 5 year follow-up. Another group reporting on the BALANCE study confirmed findings similar to those of REPAIR-AMI, with significant improvements in LV function after 5 years [23].

Following results of the REPAIR-AMI trial which showed improved LV function with intracoronary administration of BMMNCs post-AMI, the TIME, LateTIME, and SWISS-AMI trials were developed [24,25,26]. In contrast to the REPAIR-AMI trial, they concluded that BMMNC therapy did not contribute to improvements in LV recovery. The SWISS-AMI trial showed no change from baseline at a 12 month follow-up on cardiac MRI in measures of LVEF, LV volumes, and LV scar size between treatment groups (administration of intracoronary BMMNCs either 5–7 days or 3–4 weeks post-PCI) [26]. LateTIME showed no change in LVEF or wall motion between the treatment group (intracoronary stem cell administration at 2–3 weeks post-PCI) and the placebo group [25]. Most recently, in 2018, 2 year follow-up results from the TIME trial showed no improvement in LV recovery (including LV function, volumes, or infarct size measured with cardiac MRI) when comparing the treatment group (patients receiving stem cell administration on day 3 or 7 post-PCI) to the placebo [24]. These trials have shown that, regardless of timing of cell delivery, BMMNC therapy did not result in improved LV recovery when compared with a placebo group.

Overall, results of trials using BMMNC therapy have been mixed. Although results from meta-analyses (including those mentioned above) have shown promising results, numerous clinical trials have not confirmed those conclusions.

The inconsistency in outcomes from treatment with BMMNCs can be attributed to several factors. First, BMMNCs are a heterogeneous population, and it is thus difficult to determine which cell populations led to the clinical effects, or lack thereof. For example, some studies have demonstrated significant myocardial regeneration post-infarction with HSC treatment, and no effects with BMMNCs [13,27]. Additionally, a number of publications have aimed to compare methodologies between trials, concluding that apparently minor differences in isolation procedure (leading to varying ratios of HSC to MSC populations in samples); preservation protocols; quantity, quality, and timing of cell delivery; study endpoints; patient characteristics (age, comorbidities, cardiac risk factors); and the unpredictable course of AMIs can have major impacts on stem cell function and subsequent clinical outcome [28,29]. Some studies have demonstrated that samples from younger patients and increased numbers of CD34+ hematopoietic cells were independent predictors of therapeutic response, highlighting the importance of the cell product [30].

A meta-analysis from Nowbar et al. reported on the degree of discrepancy in study outcomes by analyzing specific differences found throughout 49 clinical trials that used BMSCs for cardiac repair [31]. The group concluded that studies reporting the most significant improvement in LVEF were also the studies found to have the most discrepancies, whereas studies reporting no therapeutic effect possessed little discrepancies. Although there have been a number of clinical trials evaluating BMMNC therapy in MIs (including the abovementioned BOOST, REPAIR-AMI, and BALANCE studies, as well as the BONAMI [32], REGENERATE-AMI [33], SCAMI [34], and MI3 trials [35]), due to the lack of standardization of therapies, it is difficult to draw meaningful conclusions.

Presently, the ongoing phase 3 BAMI trial (NCT01569178) is aiming to provide more conclusive data on the efficacy of BMMNC therapy in AMI. It is the largest trial to date, aiming to standardize cell collection, handling, and delivery methods. Researchers are aiming to determine if their therapeutic method can result in a 25% reduction in mortality, which is their primary endpoint. The trial is scheduled to conclude in fall 2019.

In addition to the ongoing research into the effectiveness of BMMNC therapy, given cell heterogeneity, studies isolating subpopulations of BMSCs have also emerged. These include trials using CD34+/CD133+ progenitor cells from hematopoietic lineages, or MSCs [36,37].

### 3.3. Hematopoietic Stem Cells

HSCs are a population of blood-forming cells that exist in small amounts in the bone marrow, with estimations that only 1 in 10,000 marrow cells are hematopoietic [38]. However, through identifying specific surface markers, they can be successfully isolated from the larger pool of BMSCs [7]. In initial studies on BMSC therapy, it was shown that HSCs possessed c-kit (the receptor for stem cell factor) as a surface protein, which was thought to be responsible for their ability to repair infarcted myocardium [13,27].

HSCs are characterized by the presence of surface markers CD34, CD45, CD133, and CD117 (also known as c-kit), and to date there have been a few initial clinical trials using bone-marrow-derived CD34+ or CD133+ cells [37,39,40,41]. The COMPARE-AMI trial tested intracoronary administration of CD133+ hematopoietic progenitor cells versus placebo in AMI patients with LV dysfunction [37]. The procedure was shown to be safe and feasible, and 4 month echocardiography showed superior LVEF increase in the treatment group versus the placebo group. The largest study to date has been the REGENT trial which aimed to compare the effectiveness of BMMNCs versus hematopoietic cells (CD34+) in patients with AMI [41]. Patients were randomized to one of two treatment groups (BMMNC, CD34+) or the control group. The primary study endpoint was change in LVEF and volumes, with results showing no significant differences between treatment groups (both saw a 3% increase in LVEF from baseline at the 6 month follow-up, compared with no change in the control group, and no significant changes in LV end diastolic or end systolic volumes were found). Study limitations included the lack of a placebo group, high dropout rate, and lack of pre and post study imaging for many patients. In order to derive more meaningful conclusions as to the potential for HSC therapy in patients post-MI, further clinical trials with more rigorous trial designs are needed.

Though the primary source of HSCs is the bone marrow, CD34+ cells have also been found in other tissues including peripheral blood [42], and there have been developments in the use of HSC mobilization from the marrow to the bloodstream for ease of extraction. In a 2011 phase II clinical trial assessing the efficacy and safety of intracoronary CD34+ cells for myocardial ischemia, granulocyte colony stimulating factor (G-CSF) was used to mobilize CD34+ cells from the bone marrow to peripheral blood where they could then be extracted/separated from the patient sample [43].

### 3.4. Mesenchymal Stem Cells

MSCs are a non-hematopoietic population of bone marrow cells. They are multipotent—able to differentiate into a limited number of cell types of mesenchymal lineage, including osteocytes, chondrocytes, adipocytes, myocytes, and marrow stroma [44]. MSCs are distinguished from other cell types, including HSCs, based on the presence of surface markers CD73, CD105, CD29, CD44, and CD90, as well as their lack of CD34 and CD45 surface markers which characterize HSCs [45].

Studies have shown the ability of MSCs to engraft into host tissue, rapidly differentiate into vascular endothelial and cardiomyocyte-like cells, recruit endogenous stem cells, and improve LV function post-MI [44,46,47,48]. For example, the 2009 Prochymal phase I trial showed improved global symptom scores and LVEF in the group treated with intravenous MSCs compared with in the control group [36]. Currently, there are a number of planned/ongoing clinical trials (including PROCHYMAL II (NCT00877903), MI-NSTEMI (NCT02277613), RELIEF (NCT01652209), ESTIMATION (NCT01394432)), as well as completed studies with results yet to be published, whose data will contribute towards gaining a better understanding of the future potential for the clinical application of MSC treatment [48,49].

Although results from initial studies have been encouraging, there remain challenges that must be overcome. First, post-transplant cell engraftment and survival rate is poor; this is hypothesized to be due to the hostile environment of the ischemic/infarcted cardiac tissue [50]. Thus, future research must focus on techniques to enhance long-term efficacy. Additionally, the mechanism by which MSCs mediate tissue repair is also not entirely clear. Given findings of improved cardiac function despite poor differentiation and low cellular retention, evidence suggests that the therapeutic effect of MSCs lies in supporting and facilitating endogenous repair processes via paracrine signaling [46,51]. Paracrine actions of transplanted MCSs promote numerous restorative processes including activation of resident or remote endogenous stem cells, prompting cell homing, secreting immunomodulatory and trophic factors, stabilization of the extracellular matrix, and enhanced vascularization [52].

Other studies have suggested that perhaps the hypoxic environment of infarcted tissue induces expression and release of growth factors that support angiogenesis, trigger proliferation and migration of cardiac progenitors, and promote MSC differentiation into cardiomyocytes. Factors such as vascular endothelial growth factor (VEGF), hepatocyte growth factor (HGF), and insulin-like growth factor (IGF) have been implicated in the upregulation of cardiomyocyte genes to direct differentiation and promote other reparative processes [53,54,55].

Research into the use of MSCs is growing due to a couple of key properties. First, their ease of isolation, expansion, and in vitro genetic modification makes them promising therapy candidates [46]. Next, their primary advantageous feature lies in their immunomodulatory/immunoprivileged abilities. They have the capacity to avoid rejection, a capacity that has been demonstrated in studies of both allogenic and xenogenic immunocompatibility-mismatched recipients of donor cells [56].

Theories as to the mechanisms of this immunotolerance include the MSCs being hypoimmunogenic, immunosuppressing the local environment, modulating the T-cell phenotype, and lacking or expressing low cell-surface major histocompatibility complex (MHC) antigens, contributing to decreased alloimmune response [57,58]. These properties give MSCs the capacity to act as universal donor cells for allogenic therapy, and since cells are easily expanded in vitro from moderate amounts of bone marrow aspirate, there is potential for use as an “off-the-shelf” therapy [58]. An “off-the-shelf” bank of cells would be of great benefit owing to the optimal window of time that exists post-MI where stem cell treatment exerts a therapeutic effect [19].

Another advantage of MSCs is their ease of isolation from numerous tissues. MSCs traditionally isolated from bone marrow have subsequently been isolated from adipose tissue (subcutaneous or cardiac), umbilical cord blood, Wharton’s Jelly, peripheral blood, placenta, and muscle [46,47,59,60]. Thus, MSCs are perhaps a more practical therapeutic option.

#### 3.4.1. Adipose Tissue Mesenchymal Stem Cells

Adipose tissue comprises both adipocytes and a mononuclear cell population containing MSCs similar to bone-marrow-derived MSCs (BM-MSCs). Adipose tissue mesenchymal stem cells (ATMSCs) offer some advantages over BM-MSCs, whose harvest procedure is invasive and painful. ATMSCs offer a simpler extraction procedure with decreased pain, as well as higher yields given that the density of stem cells in adipose tissue is significantly higher than in bone marrow (5% versus 0.01%) [61].

Studies have shown ATMSCs to differentiate into cardiomyocytes at a higher percentage than BM-MSCs, as well as demonstrating greater immunomodulatory effects, showing superior inhibitory effects on CD4+ and CD8+ T cell and natural killer (NK) cell activation, and suppression of B cells [62].

Recently there have been a few clinical trials using ATMSCs for CVDs. MyStromalCell is a phase II trial evaluating the effect of VEGF (specifically, VEGF-A165) stimulated ATMSCs in patients with chronic IHD and refractory angina [59]. The Precise Trial involved the use of transendocardial injection of ATMSCs for ischemic cardiomyopathy, with initial results showing improved LV mass and motion at an 18 month follow-up [63].

Following initial uses of ATMSCs extracted via liposuction from subcutaneous adipose tissue, it was hypothesized that cardiac adipose tissue mesenchymal stem cells (cATMSCs) might have an improved capacity to support cardiac homeostasis and tissue renewal. Studies have shown that cATMSCs extracted from the base of the heart and around the aortic root display an innate cardiac-like phenotype [64]. A 2014 study compared adipose tissue of cardiac versus subcutaneous origins, with pericardial-adipose-derived stem cells demonstrating superior reparative capabilities including increased myogenesis, vasculogenesis, and expression of cardiogenic transcription factors [60].

The main challenge in using these cells is the invasive harvesting procedure, and the fact that they are not readily available in comparison to non-cardiac adipose tissue stem cells. To date, cardiac adipose tissue biopsies have been obtained from patients who underwent cardiothoracic surgery prior to CABG. Additionally, typical donors tend to be older with various comorbidities that contribute to poor cell quality [64].

However, these challenges have the potential to be overcome in the future. Since a significant number of routine cardiac interventions take place in most large centers, this opens the door for the possibility of these patients becoming donors. With little added risk to the planned procedure, cardiac adipose tissue biopsy can be obtained and contribute to creating an “off the shelf” bank of cATMSCs for use in patients post-MI [46].

#### 3.4.2. Wharton’s Jelly Mesenchymal 

Wharton’s Jelly is a continuum of gelatinous mucous tissue in the umbilical cord whose role is to provide support/protection to umbilical vessels. Wharton’s jelly derived mesenchymal stem cells (WJMSCs) are a primitive population of multipotent stromal cells originating from embryonic and/or extraembryonic mesodermal tissue at day 13 of embryonic development [65,66]. Wharton’s jelly is easily extracted postnatally. WJMSCs retain many of their ESC and MSC markers in culture, and they are thought to have greater potential for cardiac differentiation compared to sources of autologous stem cells [65,66]. Through the presence of cardiogenic transcription profiles, they have been induced to express cardiac markers such as a-actin, troponin T, and connexin-43 in vitro [67,68]. Like other sources of MSCs, WJMSCs promote regeneration via paracrine mechanisms through the secretion of significant amounts of angiogenic, anti-apoptotic, and growth factors, and can be induced to differentiate into cardiomyocytes and endothelia with the ability to integrate into ischemic tissue and improve cardiac function [67,68].

In 2015, the WJ-MSC-AMI trial presented results of the first study evaluating safety and efficacy of intracoronary allogeneic WJMSC transplantation in patients with ST-elevation MI (STEMI) [47]. The trial involved introducing WJMSCs or placebo via intracoronary infusion into the infarct-related artery between 5 to 7 days following reperfusion therapy for acute STEMI. Results showed improved myocardial viability (measured by PET scan) and cardiac function, including improved infarct area perfusion, significantly improved LVEF (7.9 ± 0.9% compared to baseline), slightly reduced infarct size, and prevention of adverse LV remodeling at an 18 month follow-up (demonstrated by changes in LVEDV and LVESV). These findings are in contrast to some studies with BMMNCs that have shown a limited effect on post-MI ventricular remodeling, with no significant impact on end diastolic volumes [16,19].

Additionally, there were no signs of immune response, ectopic tissue, or tumor-associated antigens found after cell transplantation (even though they possess some embryonic stem cell markers), and their use is noncontroversial, highlighting the advantage of WJMSCs over induced pluripotent and embryonic stem cells (which will be discussed below) [47].

#### 3.4.3. Umbilical Cord Blood Mesenchymal 

Umbilical cord blood (UCB) has been considered the most abundant source of stem cells for numerous clinical purposes. Although previously used mainly in the treatment of hematological conditions, uses have expanded to effectively treat non-hematopoietic conditions as well [46]. Umbilical cord blood mesenchymal stem cells (UCBMSCs) comprise one of the non-hematopoietic stem cell populations in UCB, and although cells have failed to successfully differentiate into cardiac lineages, studies have demonstrated their role in angiogenesis and immunogenicity (as with other MSC populations) [69].

UCB extraction is safe and painless, and UCBMSCs are readily isolated and expanded in vitro with short culture time (2 days), can be cryopreserved without the loss of immunoprivileged or regenerative properties, and represent an additional population of multipotent progenitor cells [70]. There is an ongoing clinical trial using intracoronary administration of UCBMSCs in patients with IHDs (SEESUPIHD trial, NCT02666391); however, in order to achieve widespread clinical use, various issues need to be overcome. Primarily, there is a need for developing quality and potency measures to produce a product that can achieve accreditation, and creating collaboration agreements with UCB banks, particularly to increase availability of small-volume UCB units that are usually discarded [71].

#### 3.4.4. Cardiopoietic Mesenchymal Stem Cells

One of the more recent developments in the area of MSC therapy is the use of cardiopoietic MSCs. Cardiopoiesis is a process of conditioning that improves the regenerative ability of autologous stem cells via methods of lineage specification [72]. The induction of a cardiopoietic state in MSCs is designed to ensure their regenerative benefit.

The C-CURE trial was the first study to employ the use of cardiopoietic cells to treat post-MI ischemic heart failure [72]. Participants were randomized into a control group (*n* = 15), or a cell therapy group (*n* = 21) who received intramyocardial administration of bone-marrow-derived C3BS-CQR-1 cardiopoietic cells. Though primary endpoints were safety and feasibility measures rather than therapeutic effects, the treatment group showed improvements in LVEF, LVESV, and 6 minute walk test at a 6 month follow-up. Results showed the process to be as safe and feasible as non-lineage-guided BMSCs, with the addition of favorable effects on LVEF, remodeling, and overall patient wellness when compared with unguided BMSCs or standard clinical care.

Following these initial results from C-CURE, the CHART trial was designed to assess the therapeutic benefits of C3BS-CQR-1 cells in patients with chronic HF secondary to IHD, with the aim to validate cardiopoietic stem cell therapy [73]. CHART randomized 240 patients to receive either intramyocardial autologous cardiopoietic cells or placebo. The primary efficacy endpoint is a combination of mortality, worsening HF, Minnesota Living with Heart Failure Questionnaire score, 6 min walk test, LVESV, and LVEF at a 9 month follow-up. Safety endpoints include mortality, readmissions, and serious adverse events at 12 and 24 month follow-ups. The trial concluded in 2017, and final results have yet to be published.

These trials provide baseline research and insight that highlight the potential for a lineage-specified stem cell therapy without needing heart tissue itself as the cell source. This would be of significant clinical benefit given the challenges with obtaining cardiac stem cells, which will be further discussed below.

### 3.5. Embryonic Stem Cells

Embryonic stem cells (ESCs) are a population of pluripotent cells that arise from the inner cell mass of the blastocyst during embryonic development in mammals. They can give rise to any/all adult cell types, and thus have the potential to regenerate lost myocardium [74].

A primary advantage of ESC transplantation is in their capacity to differentiate into cardiomyocytes that are able to electrically integrate with cardiac muscle. For example, an early study in a swine model with AV block resulted in reversal of the block after human-ESC-derived cardiomyocytes were transplanted [75]. Furthermore, the pluripotency of ESCs gives them advantages over multipotent adult-tissue-derived stem cells which have more limited differentiation capacity.

An initial challenge with ECS studies was achieving sufficient amounts of pure cell samples from heterogeneous cell populations [76]. Strategies to overcome this limitation have included specialized gene modification, cell treatment with various biological/chemical factors, and culture methods [77].

The first clinical use of human ESCs in cardiac patients took place in 2015. The ESCORT trial delivered ESC-derived cardiac progenitor cells to patients with advanced IHD while undergoing CABG or mitral valve procedures [78,79]. Expanded cells were integrated into a fibrin patch, which was placed on the heart within a pouch/pocket created by suturing a harvested portion of the patient’s pericardium around the borders of the infarct zone. The authors report feasibility of all aspects of the procedure, and results demonstrated symptomatic improvement as well as new contractility present on echocardiographic examination, with an improved LVEF of 10% (change from 26 to 36%) from baseline at a 3 month follow-up.

Besides presenting the first application of embryonic cells in human cardiac regenerative therapy, the technique for cell transfer offered additional novelties. Previously, cell transfer had been accomplished by transepicardial injections, or percutaneous intracoronary or endoventricular catheter-based administration. Advantages of the patch-based approach include improved cell retention and survival, decreased cellular damage, decreased risk of ventricular arrhythmias, and improved patient survival and heart function preservation [80].

This initial human trial demonstrated technical feasibility and safety, thereby providing a foundation for the development of future trials that are adequately powered to evaluate efficacy [79]. Although the ESCORT trial demonstrated promising initial results, an important consideration in developing future trials is the risk of arrhythmias. Although none of the six patients in ESCORT developed arrhythmias, nonfatal ventricular arrhythmias were observed in a 2014 preclinical study using nonhuman primate models [81]. However, this primate study showed significant cell engraftment and resultant remuscularization of infarcted tissue, as well as successful electromechanical coupling of graft and host cells. These results give rise to the possibility of similar beneficial outcomes in humans if the risk of arrhythmias can be overcome.

Other considerations in future research include the challenges precluding further use of ESCs in humans at this time, which involve ethical, political, and regulatory dilemmas. Though some nations such as the USA allow the research use of embryos discarded after in vitro fertilization procedures, strict laws in other nations prohibit such use [82].

### 3.6. Induced Pluripotent Stem Cells

Prior to the last decade, the process of cellular differentiation was thought to be irreversible, an idea that was challenged in 2006 when researchers induced a pluripotent state in somatic cells [83]. Induced pluripotent stem cells (iPSCs) represent a cell population derived from various adult somatic tissues that are already specialized/differentiated. Through introduction of specific genes (traditionally via retroviral mechanisms), cells can be reprogrammed to revert back to a pluripotent, embryonic-like state. The first application of this principle involved reprogramming adult mice fibroblasts into ESC-like cells via the presence of genetic transcription factors, namely Oct3/4, Sox2, Klf4, and c-Myc [83].

As a result of the ethical and availability issues associated with ESCs, the use of iPSCs has become of clinical relevance for cardiac regenerative therapy, and initial mice studies were soon followed by the generation of iPSCs from human fibroblasts [84,85].

To be of therapeutic benefit, human iPSCs must be able to either signal angiogenesis, or differentiate into cells of cardiac lineage, and various techniques similar to those in mice have been described [86]. In vitro, cardiac progenitor cells generated from human iPSCs have shown to express cardiac proteins (including connexin-43 and myosin chain complexes) necessary for the development of adult ventricular myocyte phenotypes [87]. Additionally, cardiomyocytes with spontaneously beating sarcomeres and sensitivity to beta-adrenergic stimuli have been found in vitro when differentiated from mouse-skeletal-myoblast-derived iPSCs [88]. Furthermore, through examination of action potential characteristics, iPSCs (as well as ESCs) have been shown to possess the potential for differentiation into cells of ventricular, atrial, and nodal cell lineages [89]. These characteristics give iPSCs the capacity for superior integration into host cardiac tissue in comparison to transplantation with adult tissue stem cells.

Challenges involved with iPSC therapy include developing efficient procedures for inducing pluripotency, developing alternatives to traditional viral delivery systems, and the potential for tumorigenesis [6,90,91]. Recently, iPSCs have been obtained via defined chemicals without the use of a virus, genetic factors and signaling molecules can improve reprogramming efficiency, and to avoid teratoma formation, cells can first be directed in vitro towards differentiation into cells of cardiomyocyte lineage [6,92,93].

Although results from initial preclinical studies have shown successful outcomes on cardiac function, confirmation of in vivo safety and functionality is required prior to translation into clinical practice. Further research aims to focus on optimizing and creating standardized and reproducible differentiation techniques, and establishing clinical trials to confirm the safety and efficacy of published findings [85,89,91].

### 3.7. Cardiac Stem Cells

Although several forms of cell therapy have been beneficial to various degrees in improving cardiac function post-MI, they are not natural residents of cardiac tissue, and it is controversial as to whether they can truly regenerate lost myocardium [93]. Cells of cardiac origin have the potential to possess this capacity. Cardiac stem cells (CSCs) are a heterogeneous cell population and represent a purer source of cells with the capacity to more effectively differentiate into cardiomyocytes [94]. CSCs have typically been isolated from atrial appendages, pericardial adipose tissue, or epi/endomyocardial biopsies [6,93].

Since 2003, when Beltrami and colleagues [95] described the presence of self-replicating, clonogenic, multipotent c-kit+ cells in the heart that could give rise to cardiomyocytes, smooth muscle cells, endothelial cells, and angiogenic cells, various studies have shown that there exists a subpopulation of various classes of CSCs. In addition to ckit+ cells, other types of cardiac progenitor cells include cardiosphere-derived cells (CDCs), Sca-1+ cells, Isl1+ cells, IGF1R+ cells, cardiac side population cells, cardiac mesangioblasts, and epicardial progenitors, all of which express varying but overlapping surface markers [96].

When compared with other adult SC types, CSCs have been shown to more efficiently express cardiac markers and more effectively differentiate into cardiomyocytes in in vitro as well as in vivo animal MI models [97]. A 2016 systematic review and meta-analysis by Samanta et al. presented data on 80 placebo-controlled animal studies of preclinical CSC therapy after MI [93]. Pooled outcome analysis showed a 10.7% improvement in LVEF compared to controls, and improvements were independent of CSC type, comorbidities, and methods of cell culture used.

An advantage of CSC, similar to that of MSCs, is in their immunomodulatory and immunosuppressive abilities. Studies have shown that human CDCs express MHC class I, but lack MHC class II antigens, as well as CD80/86 costimulatory molecules, avoiding immunologic rejection in both in vitro and in vivo animal models [98]. Malliaras 2012 showed that when CDC cells were transplanted into MHC mismatched rat MI models without immunosuppression, only a mild local immune reaction took place, concluding that allogenic CDC transplantation without immunosuppression is safe, and it leads to improved cardiac function by stimulating endogenous repair processes. In vitro, allogenic CDCs led to negligible inflammatory cytokine secretion or lymphocyte proliferation. In vivo, although allogenic CDCs were found to elicit a mild inflammatory response, it was transient and local, with no evidence of rejection or systemic immune reactions.

In another study aiming to analyze the mechanisms underlying the immunologic properties of human cardiac progenitor cells (hCPCs), authors concluded that they possess a weak immunologic profile, with programmed death ligand-1 (PD-L1) responsible for the immunomodulation [99]. Authors note that the role of PD-L1 in regulating allogenic hCPC behavior could be of important benefit in the clinical setting by using its expression to specifically identify/select cells best suited for cardiac repair. Also of note, it was found that hCPC can also modulate an ongoing inflammatory T cell response, which is important when considering that transplanted cells are introduced into an inflammatory environment within the post-MI myocardium. Therefore, the introduction of CPCs to the post-MI myocardium would not contribute to further inflammation; rather, it would participate in its mitigation. The mechanism behind this action was attributed to interferon-γ (IFNγ) actions. Although it is a pro-inflammatory cytokine that upregulates MHC class II molecules on hCPCs, leading to T cell activation, it also increases expression of PD-L1—an immune regulator costimulatory molecule.

In humans, studies have tested the use of c-kit+ and CDCs, and initial clinical trial results have been encouraging. The SCIPIO phase I clinical trial showed improved LVEF and decreased infarct size at 4 and 12 month follow-ups post intracoronary administration of autologous c-kit + CSCs in ischemic cardiomyopathy patients undergoing CABG [94]. In the phase I CADUCEUS trial, autologous CDC injection post-MI into the infarct-related artery increased regional contractility and viable heart tissue, while decreasing scar mass at the 6 month MRI follow-up, though increases in LVEF were nonsignificant [100]. ALLSTAR is an ongoing phase I/II trial designed to test the safety and efficacy of delivering intracoronary allogenic CDCs in patients with LV dysfunction post-MI [101]. The trial plans to enroll up to 134 patients from up to 35 North American centers. Primary outcome measure is 30 day morbidity/mortality, and reduction in infarct size at a 1 year follow-up. CAREMI is a phase I/II trial evaluating the feasibility (safety, efficacy) of allogenic intracoronary CSC therapy post-MI [102]. Subjects included 55 patients who had AMIs with LV dysfunction and a high risk of heart failure. Although the trial has concluded, results have yet to be posted.

Challenges in using CSCs include an invasive isolation procedure, the need for costly pre-transplant ex vivo expansion to obtain adequate cell numbers for injection, and low availability [15]. To overcome the challenges of ex vivo expansion, instead of cell transplantation, there is the potential to use techniques that activate endogenous cardiac stem cells through the use of drugs, growth factors (i.e., IGF, HGF), or microRNAs [96]. Another technique to overcome challenging extraction is the use of CDCs expanded from cells obtained through percutaneous endomyocardial biopsy [103].

Since the study of CSCs is more recent than that of other stem cell sources, further analysis is needed in order to characterize cell types in terms of their surface markers, gene expression, differentiation capacities, and role in cardiac repair/homeostasis prior to further clinical application [104]. First, it is not known if cells isolated from atrial versus ventricular tissue have the potential for the same functional efficacy due to their different genetic profiles [105]. Additionally, given the significant number of subclasses of CSCs that have been discovered, it remains to be determined if these distinct classes possess distinct roles and capacities for cardiac repair. For example, though the primary source of cardiac repair in a number of studies has been c-kit+ cells, evidence showing their functional insignificance is also present, suggesting that beneficial results are due to the stimulation of capillary growth rather than cardiomyocyte generation [106].

## 4. Discussion/Future Perspectives

The discrepancies and variety in results found to date reflect the ongoing research focus, the challenges that exist in this emerging field, and the obstacles that must be overcome in order for the use of stem cell therapy in cardiovascular medicine to realize its potential.

Review articles have previously outlined in detail the factors that have contributed to the spectrum of outcomes thus far [6,15]. In 2016, the European Society of Cardiology Working Group Cellular Biology of the Heart published recommendations on how cell therapy for cardiac regeneration can be improved [107]. Authors note that future studies should aim not only to solely focus on measures of safety and efficacy, but also on more precise hypotheses around factors such as cell types (including source; allogenic versus autologous), preconditioning and culture techniques, practicality of extraction and expansion, delivery methods and timing, follow-up periods, and patient characteristics (age, health status, medical history).

Future research methodologies should aim to develop more standardized techniques so that results are comparable, and further clinical trials are needed in order to validate and build on previous findings. Following these improvements, studies that directly compare amongst/between various types of stem cells would contribute to determining the ideal source of stem cells for use in cardiac regenerative medicine. Finally, concepts of improving donor cell accessibility and developing “off-the-shelf” stem cell products should continue to be pursued, aiming to realize the potential of creating a product for convenient and timely use which will be of great clinical benefit.

## Figures and Tables

**Table 1:** Advantages, disadvantages, and clinical trials for stem cells discussed in this review.

Cell Type	Advantages	Disadvantages	Clinical Trials
Skeletal Myoblasts	Abundant, contractile abilities, withstand ischemic insult	Committed to skeletal muscle lineage	MAGIC
ESCs	Pluripotent	Ethical, political, and availability issues	ESCORT (NCT02057900)
iPSCs	Pluripotent, embryonic-like state, can be derived from various adult tissue sources, strong functional integration within myocardium	Tumorigenic if cells are not pre-differentiated, viral delivery can lead to mutations	none
BMMNCs	Most extensively studied, results show improved LV function, contractility, decreased morbidity and mortality	Heterogeneous cell population, lack standardized study methodologies, most recent trials show no improvement in cardiac function	BAMI (NCT01569178) BOOST BALANCE BONAMI (NCT00200707) REGENERATE-AMI (NCT00765453) REPAIR-AMI SCAMI (NCT00669227) MI3-Trial SWISS-AMI (NCT00355186) TIME (NCT00684021) LateTIME (NCT00684021)
MSCs	Immune-privileged, potential for allogenic use, rich source of cells (adipose in particular), readily extracted, easily expanded, variety of sources (UCB, WJ, etc.)	Lack standardized procedures, lack of long-term follow-up to determine if benefits will last	PROCHYMAL (NCT00114452) PROCHYMAL II (NCT00877903) MyStromalCell (NCT01449032) Precise Trial (NCT00426868) RELIEF (NCT01652209) ESTIMATION (NCT01394432) SEESUPIHD (NCT02666391) WJ-MSC-AMI (NCT01291329) C-CURE (NCT00810238) CHART (NCT01768702)
HSCs	Some studies show improved cardiac regeneration compared to BMSCs	Need for studies with more rigorous trial designs	REGENT (NCT00316381) COMPARE-AMI
CSCs	Superior differentiation into cardiac lineages	Invasive, low availability, costly expansion, older/autologous donors means lower quality cells	SCIPIO (NCT00474461) CADUCEUS (NCT00893360) CAREMI (NCT02439398) ALLSTAR (NCT01458405)

*ESCs (embryonic stem cells); iPSCs (induced pluripotent stem cells; BMMNCs (bone marrow mononuclear cells); LV (left ventricular); MSCs (mesenchymal stem cells); UCB (umbilical cord blood); WJ (Wharton’s jelly); HSCs (hematopoietic stem cells); BMSCs (bone marrow stem cells); CSCs (cardiac stem cells).*
